# Supplementary material for: A distal centriolar protein network controls organelle maturation and asymmetry
Source: Nat Commun. 2018 Sep 26;9:3938. doi: 10.1038/s41467-018-06286-y (PMC6158247; doi:10.1038/s41467-018-06286-y)

A distal centriolar protein network controls organelle maturation and asymmetry

Wang et al.

Supplementary Fig.1

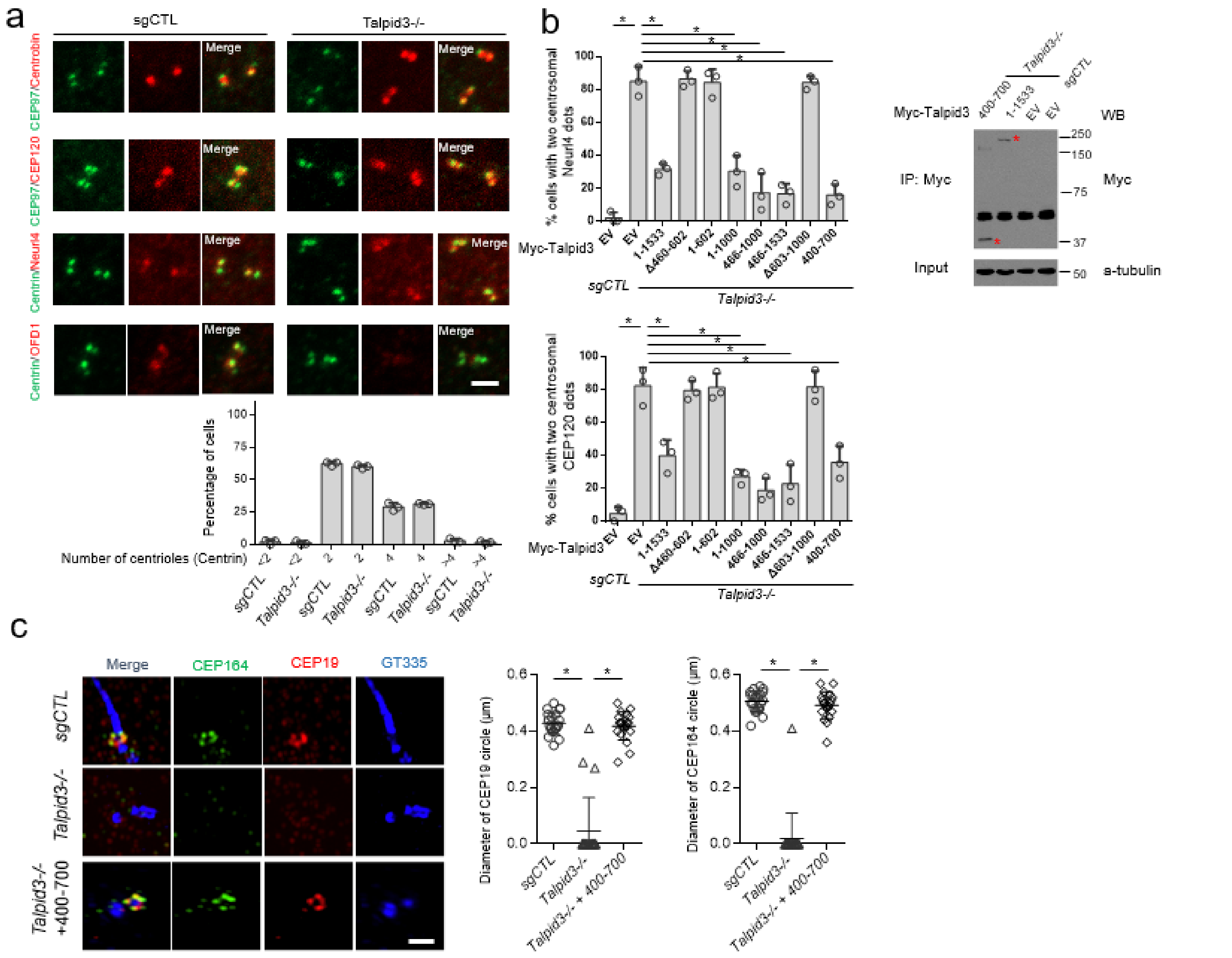

(a) Localization of DCPs and OFD1 was examined in control and *Talpid3*$^{-/-}$ RPE1 cells at S phase. Centriole number was quantified by counting Centrin dots. Scale bar =2 um. (b) Centrosomal defects in *Talpid3*$^{-/-}$ cells were rescued by infection with lentiviruses expressing different Myc-tagged Talpid3 constructs. Localization of CEP120 and Centrobin was examined by immunostaining and quantified. Specific truncation proteins were detected by western blot and are indicated by an asterisk. Cumulative data from three independent experiments are shown in (a) and (b). For each group a minimum of 100 cells/experiment was averaged. (c) Centrosomal defects of *Talpid3*$^{-/-}$ cells were rescued by infection with lentiviruses expressing Myc-tagged Talpid3 400-700 construct. Localization of CEP164 and CEP19 was examined by SIM and quantified (*N*=21). Scale bar =0.5 um. Cells were serum-starved for 24 hours and then visualized with indicated antibodies. All data are presented as *mean ± SD*. *p < 0.05 (unpaired *t-test*).

Supplementary Fig.2

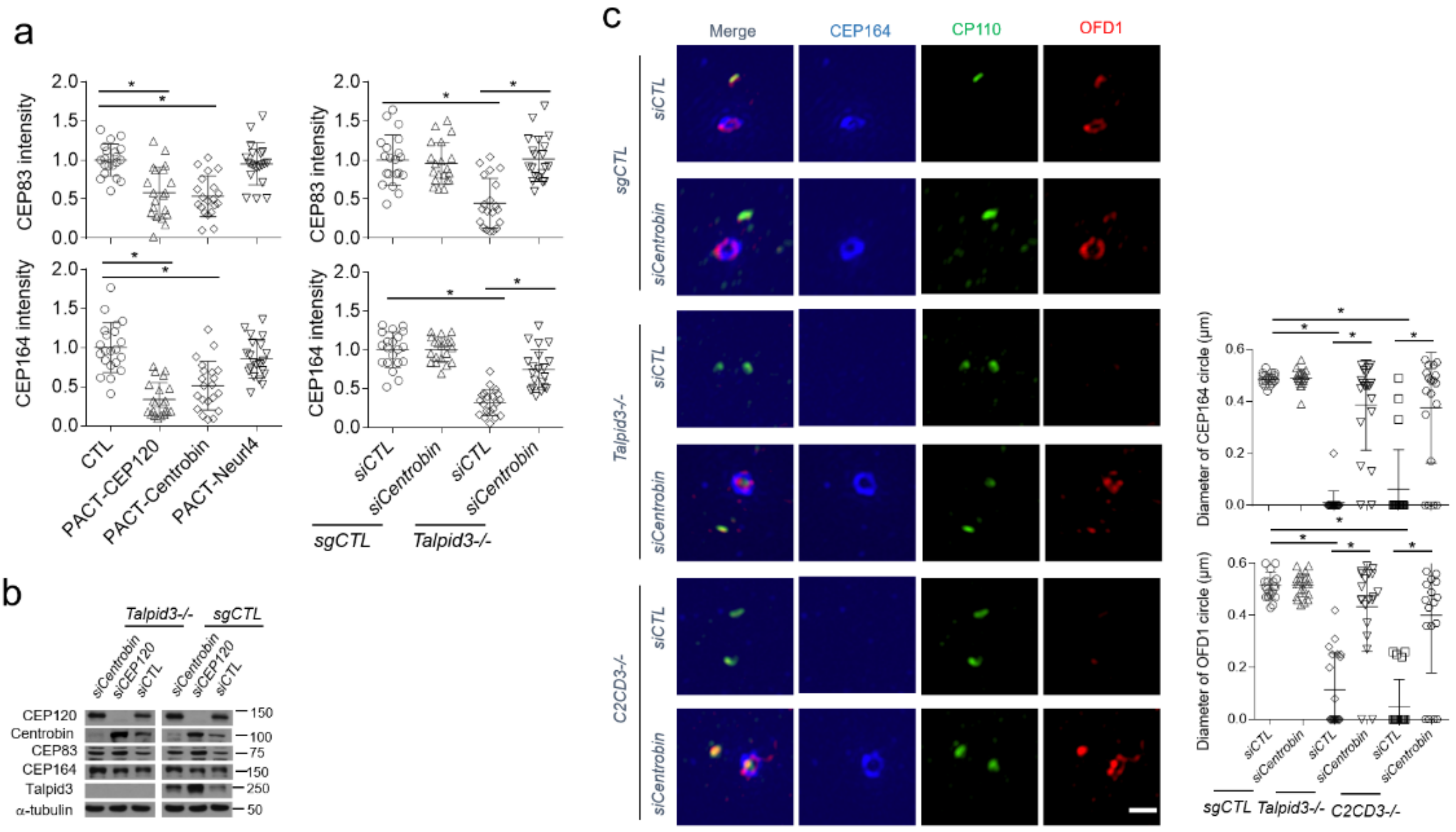

(a) Centrosomal intensity of CEP83 and CEP164 in Figure 3A and 3B was measured in G1 phase cells ($N \geq 20$). (b) Protein level of DCPs, DA proteins and Talpid3 was examined in control, and *Talpid3-/-* cells, with or without treatment of indicated siRNAs, by immunoblotting with indicated antibodies. (c) Control, *C2CD3-/-*, and *Talpid3-/-* cells were transfected with siRNAs against Centrobin. Two days after transfection, cells were serum-starved for 24 hours and were visualized with indicated antibodies using SIM. Diameter of CEP164 and OFD1 was quantified (N=20). The diameter of OFD1 on MC was measured based on co-localization of CEP164 staining, except in *C2CD3-/-* and *Talpid3-/-* cells transfected with *siCTL*, wherein MC vs DC identity cannot be identified due to lack of CEP164 staining, and in these cases, OFD1 rings on both centrioles were measured. All data are presented as *mean* ± *SD*. *$p < 0.05$ (unpaired *t-test*). Scale bar =0.5 um.

Supplementary Fig.3

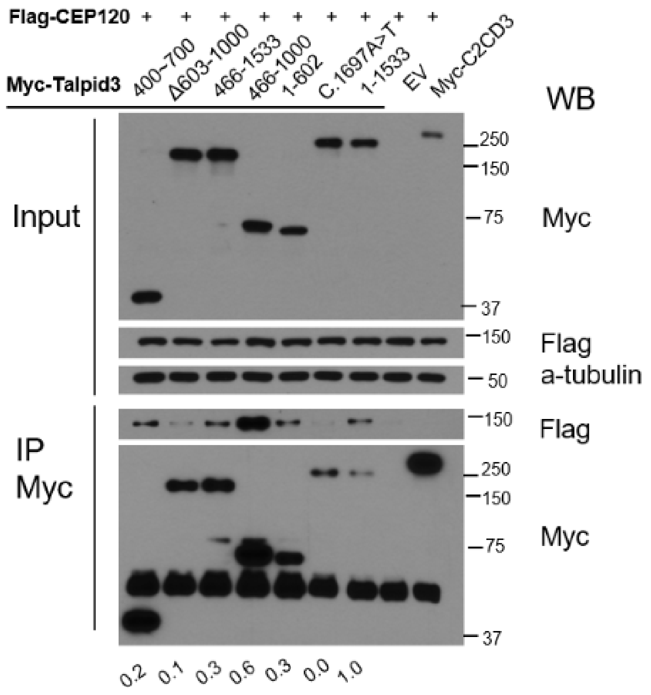

293T cells stably expressing N-terminally Flag-tagged CEP120 and N-terminally Myc-tagged Talpid3 and C2CD3 were immunoprecipitated with anti-Myc antibody. Eluates were analyzed by immunoblotting with indicated antibodies. Numbers at the bottom of each lane represent the quantification of band intensities of immunoprecipitated Flag-CEP120, which was first normalized to the lane containing the full-length Talpid3 protein (1-1533) and then normalized to the intensities of each co-immunoprecipitated, corresponding, myc-Talpid3 truncation.

Supplementary Fig.4

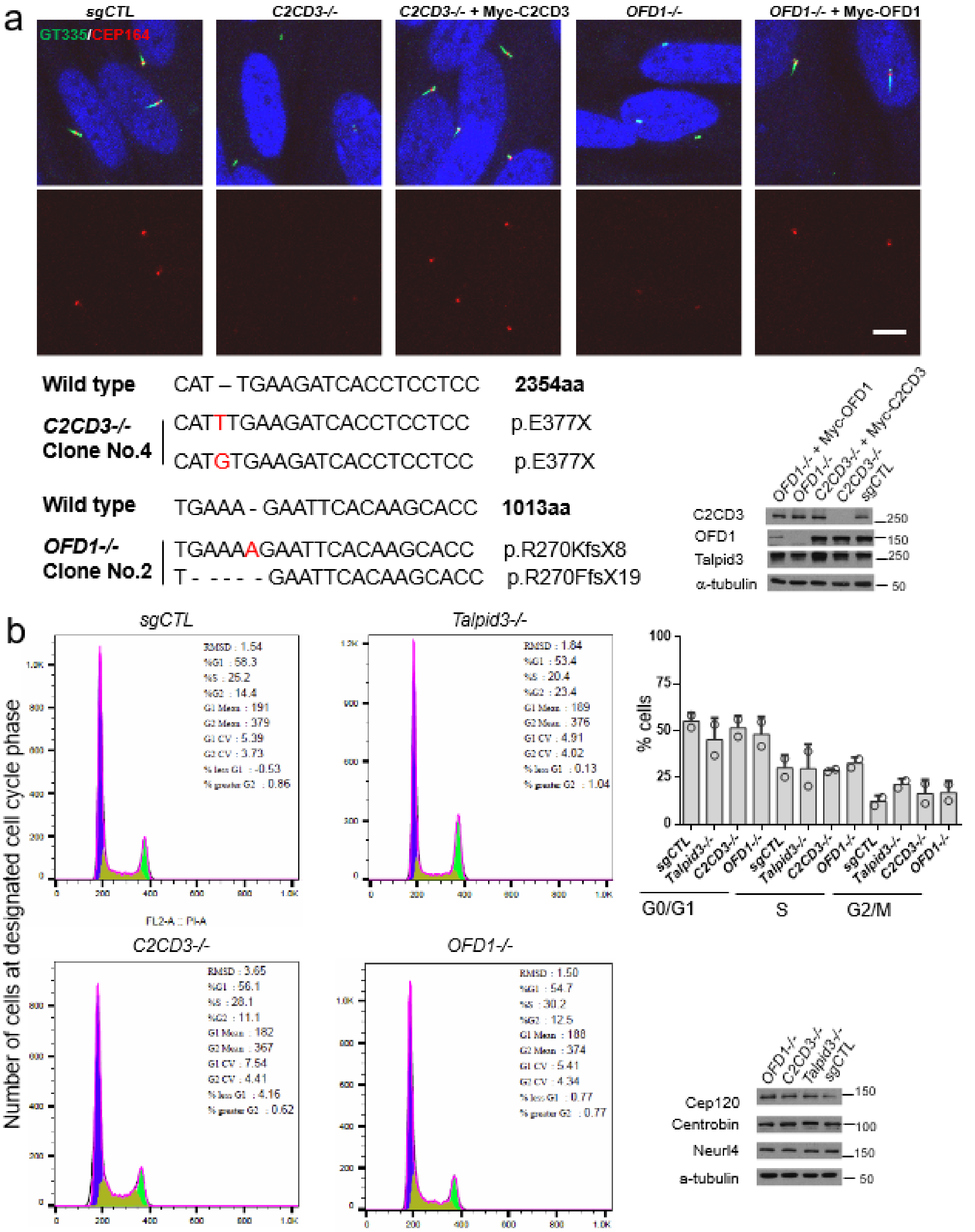

(a) Control, *C2CD3-/-* and *OFD1-/-* RPE1 cells were infected with empty, Myc-C2CD3 or Myc-OFD1 lentivirus for 72 hours, serum starved for 48 hours, and immuno-stained with antibodies against glutamylated tubulin (GT335, green), and Cep164 (green) and with DAPI (blue). Representative images are shown. Expression of Myc-C2CD3 and Myc-OFD1 was checked by immunoblotting with indicated antibodies. Bottom: *C2CD3* and *OFD1* genomic regions targeted by sgRNAs were analyzed. The sequences were compared with corresponding wild type genomic sequence. Scale bar =5 um. (b) Flow cytometry analysis of cell cycle states in control, *Talpid3-/-*, *C2CD3-/-* and *OFD1-/-* RPE1 cells. Bottom right: protein level of DCPs was examined in control, *C2CD3-/-*, *OFD1-/-*, and *Talpid3-/-* cells by immunoblotting with indicated antibodies. Data were obtained from two biologically independent experiments and are presented as *mean ± SD*. *$p < 0.05$ (unpaired *t-test*).

Supplementary Fig.5

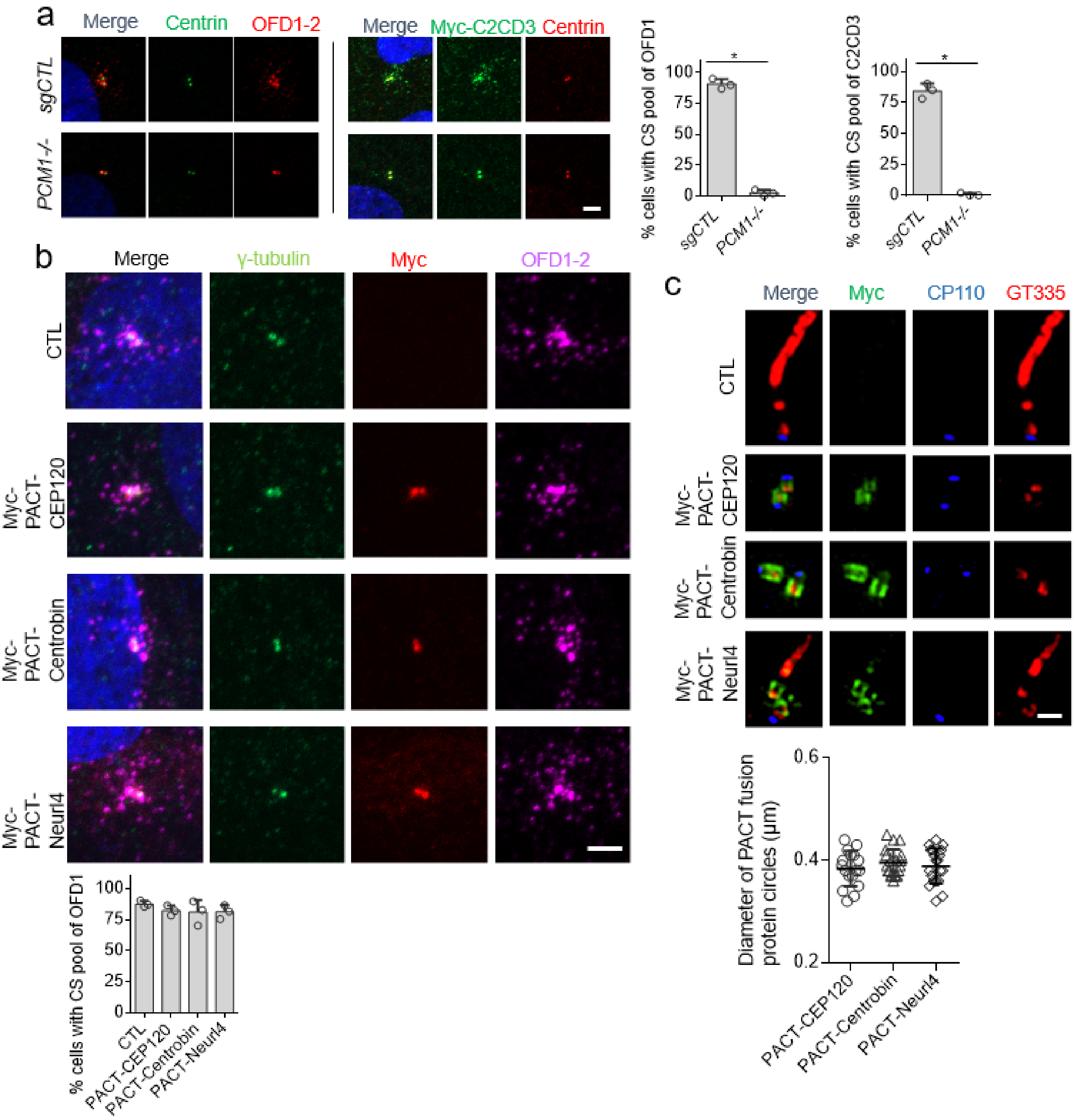

(a) Localization of CS pool of OFD1 was examined in control and *PCM1*$^{-/-}$ cells. Cells were serum-starved for 24 hours and then visualized with indicated antibodies. (b) WT RPE1 cells were transduced with lentiviruses expressing PACT-CEP120, PACT-Centrobin, or PACT-Neurl4, as indicated. Two days after transduction, cells were serum starved for 48 hours and examined by IF with indicated antibodies. Cumulative data from three independent experiments are shown in (a) and (b). For each group a minimum of 100 cells/experiment was averaged. Scale bar =2 um. (c) Localization of Myc-PACT-fused DCPs were examined by SIM. Cells were serum-starved for 24 hours and then visualized with indicated antibodies. Diameters of PACT fusion proteins are shown at bottom ($N \geq 17$). Scale bar =0.5 um. All data are presented as *mean* ± *SD*. *$p < 0.05$ (unpaired *t-test*).

Supplementary Fig.6 Uncropped western blots.

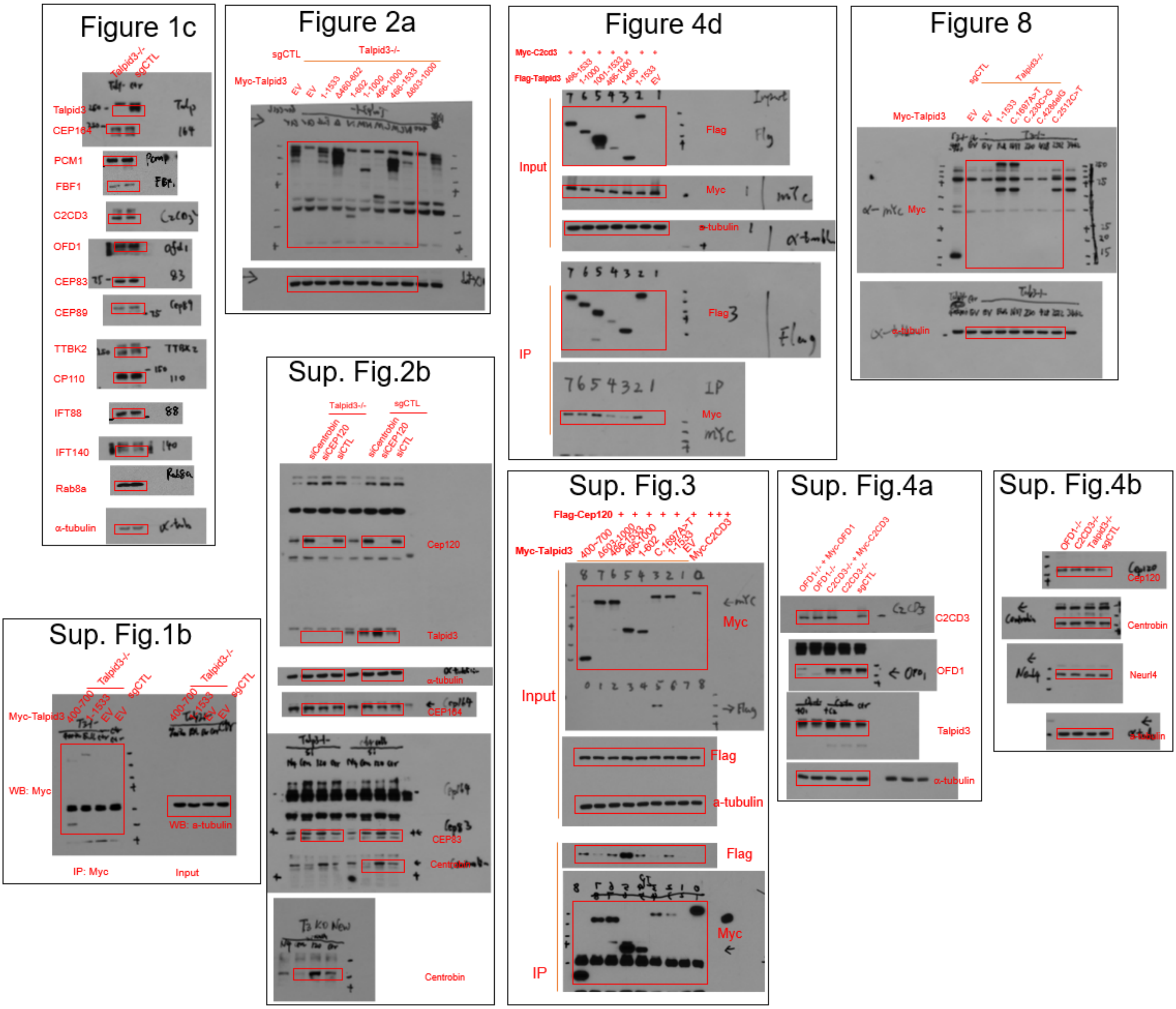

Supplement: Supplementary file 1 — Supplementary Information [file 41467_2018_6286_MOESM1_ESM.pdf]
